# On the road to robust data citation

**DOI:** 10.1038/sdata.2018.95

**Published:** 2018-05-08

*Scientific Data* is changing the way we incorporate links into our data citations. We will now be taking advantage of the resolver services offered by identifiers.org and N2T.net to provide more standardized and predictable links for biomedical datasets that have accession identifiers when they are cited in our publications.

These two parallel services help address an important problem in data citation: many of the most important community-supported biomedical repositories use accession identifier systems that must be resolved according to repository-specific linking rules. Indeed, a single repository may offer multiple link structures that all point to the same dataset. An article published today in the journal describes how identifiers.org and N2T.net, provided by the EMBL-EBI and the California Digital Library respectively, are working together to provide machine-resolvable persistent identifiers for datasets across a wide range of biomedical data repositories^1^.

These systems provide a simpler way to link published articles to datasets, and have the additional advantage of potentially being more stable over time, since they can be redirected even when individual repositories change their local linking rules.

To promote the usage of these services, *Scientific Data* will now use these links in data citations to datasets at the covered biomedical repositories, replacing the diverse repository-specific link structures we have historically used.

For *Scientific Data’s* authors, the adoption of these new services will incur no change to the submission process. These persistent link structures will be implemented by editorial staff as a part of the journal’s existing publication processes. Our authors will benefit from knowing that the dataset links in their publications will persist over the long-term. This change will also help others cite datasets that they read about at *Scientific Data* in ways that can be more easily tracked, ensuring that our authors get the credit they deserve for sharing their data.

For readers, our data citations will remain outwardly identical; these links will remain ‘hidden’ behind the accession number of the citation text according to our existing data citation formatting standards (read more: https://go.nature.com/2qBoCU6). Links in the data citations of previous publications will not be changed. We refer readers to the recent Data Descriptor by Tohge *et al*.^2^ as an example of these links in use.

In addition to helping track data reuse and assign credit for data sharing, data citation increases the transparency and provenance tracking of data that underlies research studies and findings. In 2014, the Nature Research journals endorsed the Joint Declaration of Data Citation Principles^3^, a set of principles designed to solidify the position of data as an essential, and therefore citeable, scholarly output (original announcement: https://go.nature.com/2ETEQvS). The preamble of the declaration states:

“Sound, reproducible scholarship rests upon a foundation of robust, accessible data. For this to be so in practice as well as theory, data must be accorded due importance in the practice of scholarship and in the enduring scholarly record. In other words, data should be considered legitimate, citable products of research. Data citation, like the citation of other evidence and sources, is good research practice and is part of the scholarly ecosystem supporting data reuse.”

Since endorsing this declaration, the Nature Research journals have taken a series of concrete supporting steps. One of the first was launching *Scientific Data,* a journal specifically designed to promote data as a first class research output. Each of our Data Descriptors includes a dedicated section for data citations, which are checked and curated as part of our publication process. Using the standardized links provided by identifiers.org and N2T.net will further help enhance the value of this section of our publications.

In parallel, other Nature Research journals now require data availability statements and allow datasets with digital object identifiers (DOIs) in formal reference lists^4^. Looking toward the future, Springer Nature (the publisher of the Nature Research journals) has developed a set of standard data policies^5^, and is working with other publishers and members of the research community to develop an industry roadmap for data citation^6^.

Even though formal data citations standards are still being developed, the links provided by these services can already be used by authors in their manuscripts at other journals. They can be easily included, for example, in prose data availability statements, either in addition to or instead of dataset accession numbers. And, at journals that already allow links in references lists, authors may be able to start using these immediately to reference their datasets in a more formal manner.

We will continue to review our data citation format as broader standards are agreed, but for the time being we feel this is a small but material step on the road to robust data citation.
